# Factors Associated With *Toxoplasma gondii* Infection Among Pregnant Women Admitted for Delivery in Selected Hospitals in Dar es Salaam, Tanzania

**DOI:** 10.1155/bmri/8584162

**Published:** 2026-01-05

**Authors:** Masauko Liponda, Secilia Ng′weshemi, David Munisi

**Affiliations:** ^1^ Department of Public Health and Community Nursing, School of Nursing and Public Health, The University of Dodoma, Dodoma, Tanzania, udom.ac.tz; ^2^ Department of Community Medicine, School of Medicine and Dentistry, The University of Dodoma, Dodoma, Tanzania, udom.ac.tz; ^3^ Department of Microbiology and Parasitology, School of Medicine and Dentistry, The University of Dodoma, Dodoma, Tanzania, udom.ac.tz

**Keywords:** pregnant women, risk factors, *Toxoplasma gondii*

## Abstract

Toxoplasmosis is asymptomatic in infected individuals; however, infections acquired during pregnancy pose a significant risk of the development of congenital parasitosis, leading to poor pregnancy outcomes and various degrees of chorioretinitis later in life. This study was therefore designed to determine the seroprevalence of *Toxoplasma gondii* and its associated factors among pregnant women admitted for delivery in selected hospitals in Dar es Salaam. A hospital‐based analytical cross‐sectional study was conducted among 191 pregnant women who were admitted for delivery in the selected hospitals in Dar es Salaam, Tanzania. A pretested, Kiswahili‐translated semistructured questionnaire was used to collect demographic characteristics of the respondents, as well as their risk profile and awareness on *T. gondii*. Collected blood samples were screened for *T. gondii* IgG and IgM using the FaStep TORCH IgG/IgM rapid test device as per the manufacturer′s instructions. Data analysis was done using STATA Version 14 (StataCorp, Texas, United States); figures were plotted using Microsoft Excel [2024] (Microsoft Corporation, Redmond, Washington, United States). Crude and adjusted OR were estimated by bivariable and multivariable logistic regression analysis with respective 95% CIs, respectively. A *p* value less than or equal to 0.05 was considered statistically significant. The seroprevalence of *T. gondii* among study participants was found to be 20.94%. Multivariable logistic regression for factors associated with *T. gondii* serostatus was modeled. After controlling for other factors, consumption of rodents for food (*A* *O* *R* = 10.52, 95% *C* *I* = 1.13–97.79, *p* = 0.039) and consumption of game meat (*A* *O* *R* = 3.84, 95% *C* *I* = 1.56–9.46, *p* = 0.003) were the best predictors of *T. gondii* seropositivity. Consumption of untreated water for drinking among study participants was found to be very high, although it was not associated with *T. gondii* seropositivity. This calls for the need for implementation of public health education with a particular emphasis on proper cooking of game meat and rodents before consumption and proper treatment of drinking water.

## 1. Background

*Toxoplasma gondii* is an obligate intracellular parasite responsible for causing toxoplasmosis [1]. Toxoplasmosis is a widespread zoonotic disease in the world with about one‐third of the general population being infected with the parasite [2–6]. Humans may be infected by the parasite through foodborne transmission by eating raw or undercooked meat containing cysts, through ingestion of oocysts by drinking or eating something that has been contaminated with the infective oocysts, and through congenital transmission by a woman who is newly infected with *T. gondii* during pregnancy [1]. Rarely, infections can be derived from infected blood products, tissue transplants, or unpasteurized milk, and laboratory workers who handle infected blood can also acquire infections through accidental inoculation [5].

In most cases, toxoplasmosis is asymptomatic in infected hosts, with the parasite staying latent in the victims′ tissues such as the brain; however, in immune‐compromised individuals such latent infections may take the form of severe neurological symptoms [2]. Another important group is women of reproductive age, since infections acquired during pregnancy pose a significant risk of congenital toxoplasmosis, leading to poor pregnancy outcomes such as miscarriage, serious defects of the central nervous system in the newborn, and various degrees of chorioretinitis later in life [2,7]. First‐trimester infections carry a higher risk of adverse outcomes than third‐trimester infections, but the chances of congenital transmission as a result of primary infection during pregnancy are higher if the infection is acquired during the third trimester than if it occurs during the first trimester [3, 8].

The prevention of congenital toxoplasmosis can be done through the use of drugs such as spiramycin, which is known to prevent fetal infection by more than 60% [9]. However, this would require screening during antenatal care for pregnant women, which is currently not a practice in the country [4]. *T. gondii* screening has not been part of the routine antenatal care in Tanzania mostly due to competing priorities with other infections such as HIV, malaria and syphilis, but also due to the lack of local epidemiological evidence to justify its inclusion amid competing interests [1]. Nevertheless, efforts to control *T. gondii* among pregnant women require a well‐grounded understanding of the epidemiology of the infection in this subpopulation. Despite the existence of the necessary conditions for the presence of the parasite, which includes a large stray cat population [2], the prevalence of *T. gondii* and its associated factors among pregnant women in Dar es Salaam have been insufficiently studied and remain poorly understood [3, 4]. This study was therefore designed to determine the seroprevalence of *T. gondii* and its associated factors among pregnant women admitted for delivery in selected hospitals in Dar es Salaam. This was carried out so as to accumulate enough evidence based on which antenatal screening for *T. gondii* among pregnant women will be initiated by the Ministry of Health in an effort to avert adverse pregnancy outcomes, which could be associated with the parasite.

## 2. Materials and Methods

### 2.1. Study Design and Setting

This hospital‐based analytical cross‐sectional study was conducted in Dar es Salaam region in Tanzania. Dar es Salaam Region has five districts, namely, Ilala, Kinondoni, Temeke, Kigamboni, and Ubungo, with an estimated total population of about 6.3 million people [10]. Participants were selected from three referral hospitals in Dar es Salaam Region, namely, Mwananyamala Regional Referral Hospital, Amana Regional Referral Hospital, and Temeke Regional Referral Hospital. These referral hospitals were selected based on their significance as healthcare facilities providing maternal care services in the city, attending people from all five districts constituting the Dar es Salaam city.

### 2.2. Study Population, Inclusion, and Exclusion Criteria

The study involved pregnant mothers who were admitted for delivery in the selected hospitals in Dar es Salaam, Tanzania. Specifically, the study recruited pregnant women Aged 17–43 years. Only pregnant women within the third trimester who were admitted for delivery in the selected hospitals and who were willing to participate in the study and provided informed consent or assent were selected for inclusion. Pregnant women with obstetric emergencies such as severe pre‐eclampsia and any condition that required close medical attention were excluded.

### 2.3. Sample Size and Sampling Technique

The sample size was computed using a single population proportion formula by Kish and Leslie [11] *n* = *Z* ^2^ *P*(1 − *P*)/*D* ^2^ where *n* = number of respondents needed, *Z* = is a standard normal variate which is 1.96 (for 95% ‐ confidence interval [CI]), *P* = the expected percentage of the population based on a previous study′s prevalence was 13% [12], and *d* = the desired margin of error was set at 0.05 or level of precision. By considering a nonresponse rate of 10% the minimum sample size was found to be 191 pregnant women. Systematic sampling technique was used to select study participants from each referral hospitals by first listing all eligible pregnant women who had been admitted for delivery at the start of the study to make a sampling frame. A random starting point was chosen among the first three pregnant women. Thereafter, every third woman was recruited for participation in the study. After exhausting the already admitted women, recruitment continued for newly admitted women, whereby every third newly admitted women were recruited into the study. If a selected woman did not meet the inclusion criteria (e.g., obstetric emergency), the next eligible woman was recruited to maintain the sampling interval.

### 2.4. Data Collection

#### 2.4.1. Assessment of Sociodemographic Characteristics and Risk Profile

A pretested, Kiswahili‐translated, semistructured questionnaire was used to collect information on demographic characteristics of the study participants as well as their risk profile on *T. gondii*. The questionnaire was adapted from previously published studies with slight modification to suit the current study [4, 5]. It was first developed in English, then translated into Kiswahili, and after administration, it was translated back to English by an independent translator who was blinded to the original English version.

#### 2.4.2. Blood Sample Collection and Serological Analysis

Blood samples were collected from each participant by a qualified phlebotomist. From each participant, 5 mL of venous blood was collected into a plain vacutainer tube while observing aseptic techniques. Each tube was labeled with the respective patient identification number. The collected blood was left to clot, then serum was used to run the test using the FaStep TORCH IgG/IgM rapid test device as per manufacturer′s instructions.

### 2.5. Data Analysis

A database of the collected data was created by entering the data using EpiData Version 3.1. Data analysis was done using STATA version 14 (StataCorp, Texas, United State); figures were plotted using Microsoft Excel [2024] (Microsoft Corporation, Redmond, Washington, United States). Continuous data were summarized as means and standard deviation; categorical data were summarized as proportions and percentages. Logistic regression analysis was used to determine the independent effect of the independent variables with the dependent variable by calculating the strength of the association between *T. gondii* infection and associated factors using odds ratio (OR) and 95% CI. Crude and adjusted OR were estimated by bivariable and multivariable logistic regression analysis with 95% CIs, respectively. Informed by the literature, variables were selected from the bivariable analysis for inclusion into the multivariable model. A *p* value less than or equal to 0.05 was considered as statistically significant.

### 2.6. Ethical Statement

The study received ethical clearance from the University of Dodoma Institutional Research Review Ethics Committee with Reference No. MA84/261/69/8. Permission to conduct the study was sought from and granted by the three hospitals; Amana Regional Referral Hospital permission Reference No. MoHCDGEC/ARRH/R.1/VOL II/44, Mwananyamala Regional Referral Hospital permission Reference No. MA.239/240/01/30, and Temeke regional Referral hospital permission Reference No. TRRH/RSC/9/9/02/75. Before recruitment, participants received comprehensive information on the objectives and methods of the study, their roles in the research, and the advantages and any risks associated with their participation in the study. Finally, participants signed a written informed consent form prior to their recruitment. All information collected from the participants were kept confidential and used exclusively for the study.

## 3. Results

### 3.1. Sociodemographic and Economic Characteristics of the Study Participants

A total of 191 respondents were enrolled for this study; 60 (31.41%) from Amana Hospital, 60 (31.41%) from Mwananyamala Hospital, and 71 (37.17%) from Temeke Hospital. The age of the participants ranged from 17 to 43 with a mean of 27.5 (SD 6.2). Most respondents were young to middle age mothers (20–35 years), whereas only a few were adolescents (17–19 years) and older mothers (36–43 years). The respondents were predominantly married, whereas only a few had college education (7.85%) and were formerly employed (6.8%). Almost three quarters, 142 (74.35%), of respondents belonged to the low economic status, whereas only 49 (25.65%) were of a middle economic status. Most respondents were multiparous, 138 (72.25%), whereas only 53 (27.75%) were primigravidae. Antenatal clinic attendance was relatively high, with 151 (79.06%) having 5–8 ANC attendance, whereas only 40 (20.94%) had 1–4 visits. The majority of the study participants, 145 (75.92%), reported no prior history of abortion, whereas 46 (24.08%) had experienced at least one abortion (Figure 1).

Figure 1Sociodemographic and economic characteristics of the study participants (*N* = 191).

**Figure:**
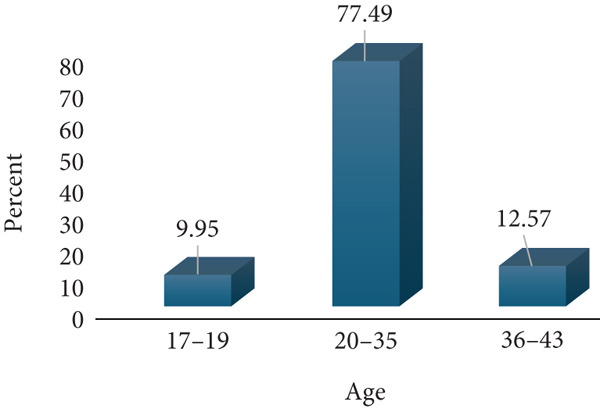
(a) Age

**Figure:**
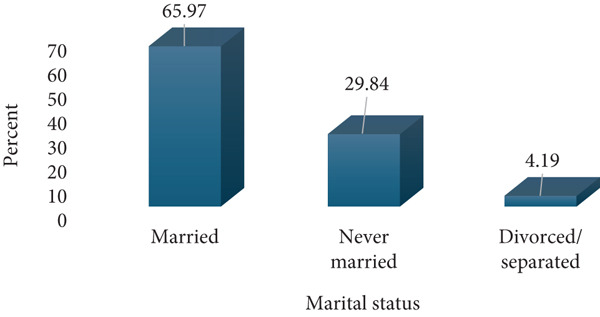
(b) Marital status

**Figure:**
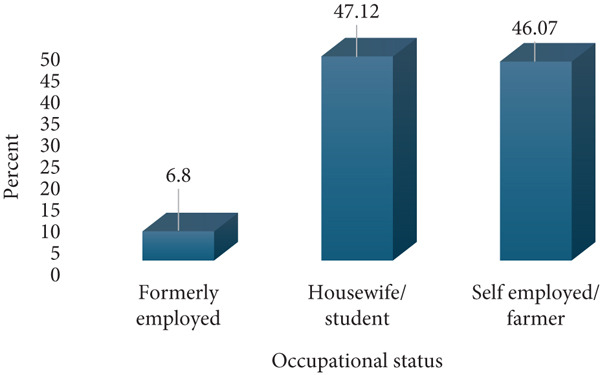
(c) Occupational status

**Figure:**
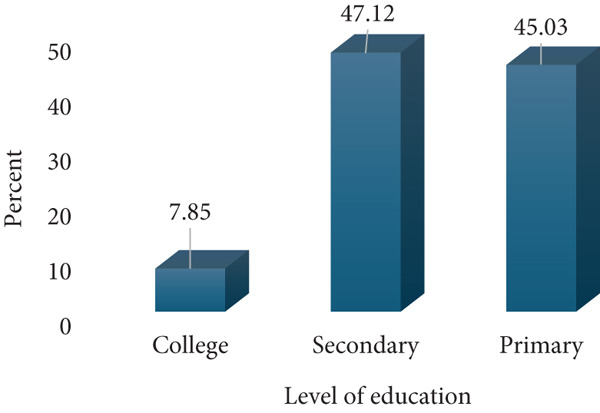
(d) Level of education

### 3.2. Prevalence of *T. Gondii* Infection Among Study Participants and Their Risk Profile

Of the 191 participants screened, 40 (20.94%) were positive for *T. gondii* IgG, whereas none of the participants tested positive for IgM. We asked respondents on various *T. gondii* risk characteristics. Although cat ownership was very low among study participants 17 (8.90%), contact with cats was quite common, whereby almost half of the mothers reported having contacts with cats 93 (48.69%). Dietary habits and water consumption practices were assessed among study participants. It was revealed that, whereas the use of untreated water for drinking was common among study participants 163 (85.34%), drinking unpasteurized milk or milk products was quite uncommon 37 (19.37%). Geophagia and salad consumption were the most commonly reported risk dietary practices among the study participants (Figure 2).

Figure 2Study participant′s *Toxoplasma gondii* risk profile (*N* = 191).

**Figure:**
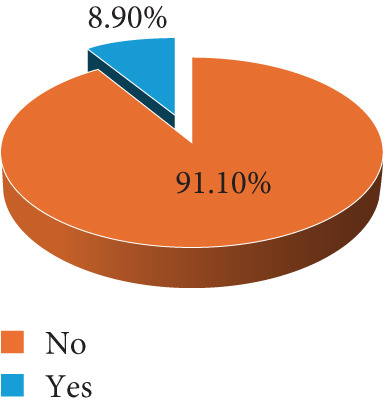
(a) Cat ownership at home

**Figure:**
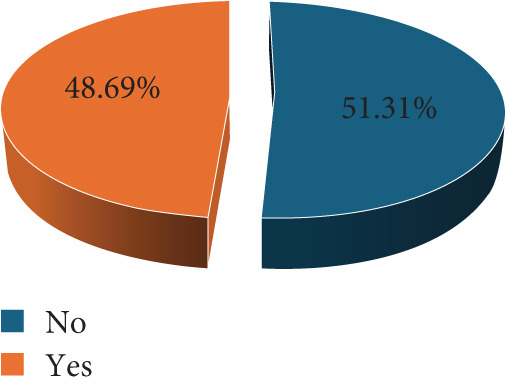
(b) Have contacts with other cats

**Figure:**
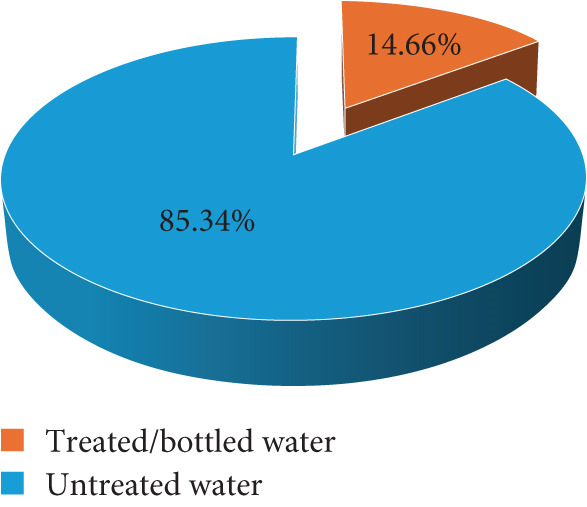
(c) Water used at home for drinking

**Figure:**
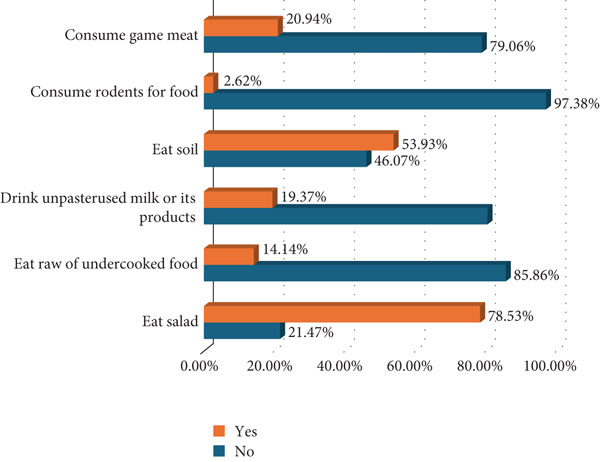
(d) Food eating habits

### 3.3. Bivariable Logistic Regression Analysis for Factors Associated With *T. gondii* Serostatus Among Study Participants

Bivariable logistic regression analysis was conducted to determine independent predictors of *T. gondii* serostatus. On bivariable logistic regression analysis, maternal age, economic status, gravidity, marital status, maternal occupation, place of residence, maternal education, number of ANC visits, history of abortion, owning cats, having contacts with other cats, consuming rodents for food, eating salad, eating raw or undercooked food, drinking unpasteurised milk or milk products, eating soil, and using untreated water at home were observed not to be associated with *T. gondii* serostatus (*p* > 0.05). However, *T. gondii* serostatus was significantly associated with consumption of game meat. Specifically, it was observed that respondents who consumed game meat were 2.5 more likely to have *T. gondii* seropositivity than those who were not consuming game meat (*p* < 0.05) (Table 1).

**Table 1:** Bivariable logistic regression analysis for factors associated with *Toxoplasma gondii* serostatus among study participants (*N* = 191).

**Variable**	**COR (95% CI)**	**p**
Maternal age		
15–19	1	
20–35	0.88 (0.27–2.84)	0.824
> 35	1.875 (0.466–7.540)	0.376
Economic status		
Middle	1	
Low	0.89 (0.40–1.94)	0.764
Gravidity		
Multipara	1	
Primigravidae	0.59 (0.25–1.38)	0.222
Marital status		
Married	1	
Never married	0.71 (0.32–1.58)	0.404
Divorced/separated	0.48 (0.06–4.04)	0.498
Maternal occupation		
Formerly employed	1	
Housewife/student	1.01 (0.26–4.03)	0.984
Self‐employed/farmer	0.74 (0.18–3.00)	0.674
Place of residence		
Urban	1	
Rural	1.24 (0.49–3.16)	0.647
Maternal education		
College	1	
Secondary	0.80 (0.20–3.18)	0.752
Primary	1.38 (0.35–5.33)	0.645
Hospital		
Amana	1	
Mwananyamala	1.11 (0.44–2.77)	0.817
Temeke	1.40 (0.60–3.285)	0.436
Number of ANC visits		
5–8	1	
1–4	0.76 (0.31–1.87)	0.548
History of abortion		
No	1	
Yes	1.06 (0.47–2.39)	0.879
Own a cat at home		
No	1	
Yes	0.79 (0.22–2.91)	0.727
Have contacts with other cats		
No	1	
Yes	1.38 (0.68–2.77)	0.370
Consume rodents for food		
No	1	
Yes	6.04 (0.97–37.47)	0.053
Eat salad		
No	1	
Yes	0.65 (0.29–1.46)	0.298
Eat raw or undercooked food		
No	1	
Yes	0.84 (0.29–2.37)	0.739
Drink unpasteurized milk or its products		
No	1	
Yes	0.53 (0.19–1.47)	0.222
Eat soil		
No	1	
Yes	0.82 (0.41–1.65)	0.576
Consume game meat		
No	1	
Yes	2.59 (1.19–5.62)	0.016∗
Water used at home for drinking		
Treated/bottled water	1	
Untreated water	0.76 (0.30–1.94)	0.569

∗Statistically significant association.

### 3.4. Multivariable Logistic Regression Analysis for Factors Associated With *T. gondii* Serostatus Among Study Participants

Multivariable logistic regression for factors associated with *T. gondii* serostatus was modeled. After controlling for other factors, consumption of rodents for food (*A* *O* *R* = 10.52, 95*%* *C* *I* = 1.13–97.79, *p* = 0.039) and consumption of game meat (AOR = 3.84, 95*%* *C* *I* = 1.56–97.46, *p* = 0.003) were the best predictors of *T. gondii* seropositivity (Table 2).

**Table 2:** Multivariable logistic regression analysis for factors associated with *T. gondii* serostatus among study participants (*N* = 191).

**Variable**	**AOR (95% CI)**	**p**
Maternal age		
15–19	1	
20–35	0.58 (0.16–2.07)	0.403
> 35	1.88 (0.42–8.43)	0.408
Economic status		
Middle	1	
Low	0.63 (0.20–1.99)	0.428
Maternal occupation		
Formerly employed	1	
Housewife/student	0.40 (0.06–2.69)	0.344
Self‐employed/farmer	0.30 (0.05–1.78)	0.184
Place of residence		
Urban	1	
Rural	0.7 (0.22–2.19)	0.538
Maternal education		
College	1	
Secondary	1.21 (0.23–6.42)	0.825
Primary	2.46 (0.41–14.61)	0.321
Own a cat at home		
No	1	
Yes	0.47 (0.10–2.09)	0.319
Have contacts with other cats		
No	1	
Yes	1.39 (0.60–3.23)	0.441
Consume rodents for food		
No	1	
Yes	10.52 (1.13–97.79)	0.039∗
Eat salad		
No	1	
Yes	0.67 (0.28–1.61)	0.374
Eat raw or undercooked food		
No	1	
Yes	0.65 (0.19–2.19)	0.490
Drink unpasteurised milk or its products		
No	1	
Yes	0.43 (0.13–1.38)	0.155
Eat soil		
No	1	
Yes	1.03 (0.43–2.44)	0.951
Consume wild animals for food		
No	1	
Yes	3.84 (1.56–9.46)	0.003∗
Water used at home for drinking		
Treated/bottled water	1	
Untreated water	0.71 (0.25–2.00)	0.519

∗Statistically significant association.

## 4. Discussion

The control of congenital toxoplasmosis would require controlling the disease among women of reproductive age, or pregnant women. Controlling *T. gondii* among pregnant women requires a well‐grounded understanding of the epidemiology of the infection in this subpopulation including understanding the seroprevalence of the infection and its predictors. We therefore conducted this study to determine the seroprevalence of *T. gondii* and its associated factors among pregnant women admitted for delivery in selected hospitals in Dar es Salaam. This study revealed an overall *T. gondii* IgG seroprevalence among pregnant women admitted for delivery to be 20.95%, none of the study participants tested positive for IgM. This prevalence indicates that the women had exposure to *T. gondii;* however, the prevalence is relatively lower than that, which has been previously reported in Dar es Salaam, which was found to range between 27.2% and 35% [13–15], Mwanza 30.9% [4], and Kilimanjaro 44.5% [16], suggesting that our observed prevalence is relatively moderate. This suggests further that the risk of exposure in the region is relatively lower when compared with other regions in the country. However, it is similar to a prevalence that was reported in Iran 20.8 [17]. This variation in seropositivity is likely to be due to variation in the geographical distribution of *T. gondii*, climatic conditions, most importantly, relative humidity and temperature, which are known to affect parasite survival in the environment, cat population densities and feeding habits, food eating habits of the human population, and socioeconomic status [18–20].

It has been reported that the prevalence of *T. gondii* seropositivity increases with increasing age, the main reason being increasing the risk of exposure with age [21, 22]. The present study has found no significant relationship between age and *T. gondii* seropositivity; however, women above 35 years had a higher proportion of infection compared with those of the other age groups. Similarly, some studies have reported a significantly higher *T. gondii* seropositivity among pregnant women living in urban settings compared with those living in rural areas [23]. However, our study found no relationship between pregnant women living in urban areas and those in rural areas in terms of their *T. gondii* serostatus. This observation is similar to that which was reported in other studies elsewhere [22, 24].

Drinking untreated contaminated water can be a potential source of *T. gondii* infection [25]. Our study has found that 85.34% of women are using untreated water for drinking at home, with only 13.66% reporting using treated or bottled water for drinking. However, this observation was not demonstrated to be significantly associated with *T. gondii* seropositivity. This was contrary to studies done in Nigeria that reported significantly higher *T. gondii* seroprevalence among pregnant women drinking well water compared with those using packed water [23]. However, the high proportion of pregnant women reporting using untreated water is of significant concern, calling for public health education to emphasize the need for drinking well‐treated water as a means of preventing the transmission of orally transmitted infections including *T. gondii*.

Animals of the feline family including domestic cat are the only known definitive hosts for *T. gondii*, and they are responsible for discharging millions of infective oocysts in their feces playing a vital role in the transmission of *T. gondii* [26–28]. Several studies have demonstrated that owning a cat at home is associated with an increased risk for *T. gondii* seropositivity among household members [3, 24, 29–31]. Our study showed no relationship between cat ownership and *T. gondii* serostatus, this is similar to another study that was carried out in Dar es Salaam in 2023 [14] and other studies conducted elsewhere [22, 23, 32]. However, it is important to understand that, the direct relationship between human toxoplasmosis and domestic cat ownership can be challenging to establish because oocysts are not found on cat fur [33], the actual and most common source of human infection is soil and not the cat themselves [34]. The parasite oocysts are shed from the cat uninfective, they sporulate and become infective outside the cat in the soil [35]. Thus, the relationship between cat ownership and human toxoplasmosis that is observed in other studies is likely to be a result of the way the cats′ litter box is cleaned rather than the simple presence of cats in the household [3].

In the maintenance and spread of *T. gondii*, rodents serve as key intermediate and reservoir hosts. When rodents are eaten by humans, as many human populations do, toxoplasmosis can be directly transmitted from rodents to humans [36, 37]. In the present study, consumption of rodents for food was observed to significantly increase the risk for *T. gondii* seropositivity. Consumption of rodents has been reported to increase the risk for toxoplasmosis in other studies [37] Similarly, this study reports an increased risk for toxoplasmosis among pregnant women who reported consuming game meat, as also reported in another study elsewhere [38]. Where very high seropositivity against T. gondii in humans, wild mammals, peri‐domestic rodents, and domestic animals have been reported, which gives an indication of the existence of a sylvatic cycle in communities that consume game meat [38].

Consumption of raw vegetables has been associated with *T. gondii* seropositivity among pregnant women owing to the possibility for the raw vegetables to host *T. gondii* oocysts that remain infective for 12–24 months under favorable conditions [14, 39]. However, our study did not find a significant relationship between the consumption of salads or undercooked food and *T. gondii* serostatus. This discrepancy is likely to be due to the differences in food eating and preparation habits, as well as the differences in other *T. gondii* risk behaviors in the studied populations. Similarly, eating soil has not been demonstrated as a risk factor for *T. gondii* infection in the present study, consistent with what has previously been reported [3] but in contrast to other studies that have demonstrated such an association [40–42].

It was plausible that the findings in this study could have been influenced by, first of all, the involvement of only three regional referral hospitals in the region, whereas there could be other pregnant women who were admitted for delivery at other lower‐level healthcare facilities across the five districts. Consequently, the recruited pregnant women may not have fully represented pregnant women from all the five districts in the region, thus limiting generalizability of the findings to the entire region. It is recommended that future studies include healthcare facilities at all levels from all the five districts of Dar es Salaam. But also, the use of questionnaires to collect risk factor data was subject to recall bias or underreporting of sensitive practices such as consumption of rodents. We recommend that future studies incorporate qualitative components such as focus groups discussions or key informants′ interviews to better understand cultural issues around food consumption in the region.

## 5. Conclusion and Recommendation

The present study has demonstrated that the prevalence of *T. gondii* among pregnant women in the study area is moderate, indicating that women had exposure to the parasite in the area, thereby providing evidence that could support the Ministry of Health in Tanzania to consider integrating toxoplasmosis screening into routine antenatal care. The study demonstrated no statistically significant differences in seroprevalence between hospitals. It is therefore recommended that, future studies with relatively larger sample size explore hospital level variations. Importantly, our study highlights consumption of rodents and game meat as independent predictors of seropositivity among pregnant women thereby contributing novel evidence for the factors associated with *T. gondii* infection to the Tanzanian context. This adds to the overall global understanding of *T. gondii* epidemiology, thereby underscoring the need for a targeted public health education on toxoplasmosis in Tanzania.

The study has further demonstrated that consumption of untreated water for drinking among study participants is very high although it was not associated with *T. gondii* seropositivity. This calls for the need for implementation of public health education campaigns on safe food practices with a particular emphasis on proper cooking of game meat and rodents before consumption. The health education should also emphasize proper treatment of drinking water so as to reduce the risk for transmission of *T. gondii* in the study population. It is further recommended that healthcare workers should educate pregnant women during antenatal care visits. Lastly, as these recommendations are implemented, further studies should be undertaken to monitor the prevalence and evaluate the implemented interventions.

## Conflicts of Interest

The authors declare no conflicts of interest.

## Funding

No funding was received for this manuscript.

## Supporting information


**Supporting Information 1** Additional supporting information can be found online in the Supporting Information section. File S1: Questionnaire for toxoplasmosis—English version.

## Data Availability

The data that support the findings of this study are available from the corresponding author upon reasonable request.

## References

[1] Gelaye W. , Kebede T. , and Hailu A. , High Prevalence of Anti-Toxoplasma Antibodies and Absence of *Toxoplasma Gondii* Infection Risk Factors Among Pregnant Women Attending Routine Antenatal Care in Two Hospitals of Addis Ababa, Ethiopia, Journal of Infectious Diseases. (2015) 34, 41–45, 10.1016/J.IJID.2015.03.005, 2-s2.0-84925654806, 25759324. 25759324

[2] Salamon D. and Bulanda M. , *Toxoplasma Gondii* and Women of Reproductive Age: An Analysis of Data From the Chair of Microbiology, Jagiellonian University Medical College in Cracow, Annals of Parasitology. (2014) 60, no. 4, 291–296, http://www.ncbi.nlm.nih.gov/entrez/query.fcgi?cmd=Retrieve%26db=PubMed%26dopt=Citation%26list_uids=25706428. 25706428

[3] Zemene E. , Yewhalaw D. , Abera S. , Belay T. , Samuel A. , and Zeynudin A. , Seroprevalence of *Toxoplasma Gondii* and Associated Risk Factors Among Pregnant Women in Jimma Town, Southwestern Ethiopia, BMC Infectious Diseases. (2012) 12, no. 1, 10.1186/1471-2334-12-337, 2-s2.0-84870332648, 23216887. PMC3519766 23216887

[4] Mwambe B. , Mshana S. E. , Kidenya B. R. , Massinde A. N. , Mazigo H. D. , Michael D. , Majinge C. , and Groß U. , Sero-Prevalence and Factors Associated With *Toxoplasma Gondii* Infection Among Pregnant Women Attending Antenatal Care in Mwanza, Tanzania, Parasites & Vectors. (2013) 6, no. 1, 10.1186/1756-3305-6-222, 2-s2.0-84881063168. PMC3750225 23915834

[5] Tenter A. M. , Heckeroth A. R. , and Weiss L. M. , *Toxoplasma Gondii*: From Animals to Humans, International Journal for Parasitology. (2000) 30, no. 12-13, 1217–1258, 10.1016/S0020-7519(00)00124-7, 2-s2.0-0033647231. 11113252 PMC3109627

[6] Bigna J. J. , Tochie J. N. , Tounouga D. N. , Bekolo A. O. , Ymele N. S. , Youda E. L. , Sime P. S. , and Nansseu J. R. , Global, Regional, and Country Seroprevalence of *Toxoplasma Gondii* in Pregnant Women: A Systematic Review, Modelling and Meta-Analysis, Scientific Reports. (2020) 10, no. 1, 10.1038/s41598-020-69078-9, 32694844. PMC7374101 32694844

[7] Paquet C. , Yudin M. H. , Yudin M. H. , Allen V. M. , Bouchard C. , Boucher M. , Caddy S. , Castillo E. , Money D. M. , Murphy K. E. , Ogilvie G. , Paquet C. , van Schalkwyk J. , and Senikas V. , Toxoplasmosis in Pregnancy: Prevention, Screening, and Treatment, Journal of Obstetrics and Gynaecology Canada. (2013) 35, no. 1, 78–79, 10.1016/S1701-2163(15)31053-7, 2-s2.0-84885461078. 23343802

[8] Dunn D. , Wallon M. , Peyron F. , Petersen E. , Peckham C. , and Gilbert R. , Mother-to-Child Transmission of Toxoplasmosis: Risk Estimates for Clinical Counselling, Lancet. (1999) 353, no. 9167, 1829–1833, 10.1016/S0140-6736(98)08220-8, 2-s2.0-0033614660, 10359407. 10359407

[9] Goldstein E. J. C. , Montoya J. G. , and Remington J. S. , Clinical Practice: Management of *Toxoplasma Gondii* Infection During Pregnancy, Clinical Infectious Diseases. (2008) 47, no. 4, 554–566, 10.1086/590149, 2-s2.0-48749095393. 18624630

[10] Mghamba J. M. , Oriyo N. M. , Bita A. A. F. , Shayo E. , Kagaruki G. , Katsande R. , Hussein A. , Kishimba R. S. , Urio L. J. , Lema N. , Camara N. , Makundi V. , Mengestu T. K. , Saguti G. E. , Habtu M. M. , Kwesi E. , Bakari M. , Mfaume R. , Makubi A. , and Subi L. , Compliance to Infection Prevention and Control Interventions for Slowing Down COVID-19 in Early Phase of Disease Transmission in Dar es Salaam, Tanzania, Pan African Medical Journal. (2022) 41, no. 1, 10.11604/PAMJ.2022.41.174.31481, 35573435. PMC9074051 35573435

[11] Kish L. , Sampling Organizations and Groups of Unequal Sizes, American Sociological Review. (1965) 30, no. 4, 10.2307/2091346, 2-s2.0-5844244390. 14325826

[12] Onduru O. G. and Aboud S. , Prevalence and Risk Factors for Typical Signs and Symptoms of Toxoplasmosis in Children Born to at Risk Pregnant Women Attending Prenatal Care in Temeke district, Tanzania, Scientific African. (2021) 11, e00690, 10.1016/J.SCIAF.2020.E00690.

[13] Kibwana U. O. , Manyahi J. , Nkinda L. B. , Renatus D. S. , Kamori D. D. , and Majigo M. , Seroprevalence and Risk Factors of Toxoplasmosis Among HIV Infected Women of Child-Bearing Age Attending Care and Treatment Clinics in Dar es Salaam, Tanzania, African Health Sciences. (2022) 22, no. 4, 470–476, 10.4314/ahs.v22i4.53, 37092062. 37092062 PMC10117500

[14] Lushina M. , Mushi V. , Tarimo D. , and Babafemi E. O. , Seroprevalence of *Toxoplasma Gondii* and Associated Risk Factors Among Pregnant Women Attending Antenatal Care in Ilala Municipality, Dar es Salaam, Tanzania, East Africa Science. (2023) 5, no. 1, 29–40, 10.24248/EASCI.V5I1.73.

[15] Doehring E. , Reiter-Owona I. , Bauer O. , Kaisi M. , Hlobil H. , Quade G. , Hamudu N. A. S. , and Seitz H. M. , *Toxoplasma Gondii* Antibodies in Pregnant Women and their Newborns in Dar Es Salaam, Tanzania, American Journal of Tropical Medicine and Hygiene. (1995) 52, no. 6, 546–548, 10.4269/AJTMH.1995.52.546, 2-s2.0-0029049792, 7611563. 7611563

[16] Paul E. , Kiwelu I. , Mmbaga B. , Nazareth R. , Sabuni E. , Maro A. , Ndaro A. , Halliday J. E. , and Chilongola J. , *Toxoplasma Gondii* Seroprevalence Among Pregnant Women Attending Antenatal Clinic in Northern Tanzania, Tropical Medicine and Health. (2018) 46, no. 1, 10.1186/S41182-018-0122-9/TABLES/3. PMC6245905 30479556

[17] Ahmadi R. , Sadeghinasab J. , Siyadatpanah A. , Mirzaei F. , Aghcheli B. , and Norouzi R. , Frequency *of Toxoplasma Gondii*, Rubella, and Cytomegalovirus Antibodies in Pregnant Women in Yazd Province, Iran, Clinical Microbiology and Infection. (2023) 10, no. 4, 145–151, 10.34172/ajcmi.3486.

[18] Tarekegn Z. S. , Dejene H. , Addisu A. , and Dagnachew S. , Potential Risk Factors Associated With Seropositivity for *Toxoplasma Gondii* Among Pregnant Women and HIV Infected Individuals in Ethiopia: A Systematic Review and Meta-Analysis, PLoS Neglected Tropical Diseases. (2020) 14, no. 12, e0008944, 10.1371/JOURNAL.PNTD.0008944, 33320848. 33320848 PMC7771857

[19] Adugna B. , Tarekegn Z. S. , Damtie D. , Woldegebreal S. N. , Raju R. P. , Maru M. , and Ayele A. , Seroepidemiology of *Toxoplasma Gondii* Among Pregnant Women Attending Antenatal Care in Northwest Ethiopia, Infection and Drug Resistance. (2021) 14, 1295–1303, 10.2147/IDR.S299106, 33883909. 33883909 PMC8053702

[20] Odeniran P. O. , Omolabi K. F. , and Ademola I. O. , Risk Factors Associated With Seropositivity for *Toxoplasma Gondii* in Population-Based Studies Among Immunocompromised Patients (Pregnant Women, HIV Patients and Children) in West African Countries, Cameroon and Gabon: A Meta-Analysis, Acta Tropica. (2020) 209, 105544, 10.1016/J.ACTATROPICA.2020.105544, 32461111. 32461111

[21] Bobić B. , Jevremović I. , Marinković J. , Šibalić D. , and Djurković-Djaković O. , Risk Factors for Toxoplasma Infection in a Reproductive Age Female Population in the Area of Belgrade, Yugoslavia, European Journal of Epidemiology. (1998) 14, no. 6, 605–610, 10.1023/A:1007461225944, 2-s2.0-0031660366, 9794128. 9794128

[22] Ertug S. , Okyay P. , Turkmen M. , and Yuksel H. , Seroprevalence and Risk Factors for Toxoplasma Infection Among Pregnant Women in Aydin Province, Turkey, BMC Public Health. (2005) 5, 10.1186/1471-2458-5-66, 2-s2.0-23244463560, 15958156. PMC1177966 15958156

[23] Shalangwa Ishaku B. , Ajogi I. , Udoudoh Umoh J. , Lawal I. , and Randawa A. , Seroprevalence and Risk Factors for *Toxoplasma Gondii* Infection Among Antenatal Women in Zaria, Nigeria, Research Journal of Medicine and Medical Sciences. (2009) 4, no. 2, 483–488.

[24] Baril L. , Ancelle T. , Goulet V. , Thulliez P. , Tirard-Fleury V. , and Carme B. , Risk Factors for Toxoplasma Infection in Pregnancy: A Case-Control Study in France, Scandinavian Journal of Infectious Diseases. (1999) 31, no. 3, 305–309, 10.1080/00365549950163626, 2-s2.0-0032772139, 10482062. 10482062

[25] Bowie W. R. , King A. S. , Werker D. H. , Isaac-Renton J. L. , Bell A. , Eng S. B. , and Marion S. A. , Outbreak of Toxoplasmosis Associated With Municipal Drinking Water. The BC Toxoplasma Investigation Team, Lancet. (1997) 350, no. 9072, 173–177, 10.1016/s0140-6736(96)11105-3, 2-s2.0-0031578297, 9250185. 9250185

[26] Murebwayire E. , Njanaake K. , Ngabonziza J. C. S. , Njunwa K. J. , and Jaoko W. , Seroprevalence and Risk Factors of *Toxoplasma Gondii* Infection Among Pregnant Women Attending Antenatal Care in Kigali, Rwanda, Tanzania Journal of Health Research. (2017) 19, no. 1, 10.4314/THRB.V19I1.2, 2-s2.0-85010377879.

[27] Abdel-Raouff M. , Mobarak Elbasheir M. , and Elbasheir M. M. , Sero-Prevalence of *Toxoplasma Gondii* Infection Among Pregnant Women Attending Antenatal Clinics in Khartoum and Omdurman Maternity Hospitals, Sudan, Journal of Coastal Life Medicine. (2014) 2, no. 6, 496–499, 10.12980/JCLM.2.2014APJTD-2014-0062.

[28] Yohanes T. , Zerdo Z. , Chufamo N. , and Abossie A. , Toxoplasma Gondii Infection: Seroprevalence and Associated Factors of *Toxoplasma Gondii* Infection Among Pregnant Women Attending in Antenatal Clinic of Arba Minch Hospital, Cross Sectional Study, Translational Biomedicine. (2017) 8, no. 1, 2172-0479, 10.21767/2172-0479.1000105.

[29] Hassanain N. A. H. , Shaapan R. M. , and Hassanain M. A. H. , Associated Antenatal Health Risk Factors With Incidence of Toxoplasmosis in Egyptian Pregnant Women, Pakistan Journal of Biological Sciences: PJBS. (2018) 21, no. 9, 463–468, 10.3923/pjbs.2018.463.468, 2-s2.0-85060032665, 30724048. 30724048

[30] Bamba S. , Cissé M. , Sangaré I. , Zida A. , Ouattara S. , and Guiguemdé R. T. , Seroprevalence and Risk Factors of *Toxoplasma Gondii* Infection in Pregnant Women From Bobo Dioulasso, Burkina Faso, BMC Infectious Diseases. (2017) 17, no. 1, 10.1186/s12879-017-2583-6, 2-s2.0-85021905729, 28693432. PMC5504642 28693432

[31] Teweldemedhin M. , Gebremichael A. , Geberkirstos G. , Hadush H. , Gebrewahid T. , Asgedom S. W. , Gidey B. , Asres N. , and Gebreyesus H. , Seroprevalence and Risk Factors of *Toxoplasma Gondii* Among Pregnant Women in Adwa District, Northern Ethiopia, BMC Infectious Diseases. (2019) 19, no. 1, 10.1186/s12879-019-3936-0, 2-s2.0-85064396190, 30991956. PMC6469075 30991956

[32] Nijem K. I. and Al-Amleh S. , Seroprevalence and Associated Risk Factors of Toxoplasmosis in Pregnant Women in Hebron District, Palestine, EMHJ-Eastern Mediterranean Health Journal. (2009) 15, no. 5, 1278–1284, 20214142. 20214142

[33] Dubey J. P. , Duration of Immunity to Shedding of *Toxoplasma Gondii* Oocysts by Cats, Journal of Parasitology. (1995) 81, no. 3, 410–415, 10.2307/3283823, 2-s2.0-0029070145, 7776126. 7776126

[34] Dubey J. P. , Sources of *Toxoplasma Gondii* Infection in Pregnancy: Until Rates of Congenital Toxoplasmosis Fall, Control Measures are Essential, BMJ. (2000) 321, no. 7254, 127–128, 10.1136/bmj.321.7254.127, 2-s2.0-0034661759, 10894674. 10894674 PMC1118145

[35] Mehlhorn H. , Toxoplasma Gondii, Encyclopedic Reference of Parasitology, 2001, Springer, 645–645, 10.1007/3-540-29834-7_1460.

[36] Galeh T. M. , Sarvi S. , Montazeri M. , Moosazadeh M. , Nakhaei M. , Shariatzadeh S. A. , and Daryani A. , Global Status of *Toxoplasma Gondii* Seroprevalence in Rodents: A Systematic Review and Meta-Analysis, Frontiers in Veterinary Science. (2020) 7, 10.3389/FVETS.2020.00461, 32851037. PMC7411222 32851037

[37] Mgode G. F. , Katakweba A. S. , Mhamphi G. G. , Fwalo F. , Bahari M. , Mashaka M. et al., Prevalence of Leptospirosis and Toxoplasmosis: A Study of Rodents and Shrews in Cultivated and Fallow Land, Morogoro Rural District, Tanzania, Tanzania Journal of Health Research. (2014) 16, no. 3, 250–255, 10.4314/THRB.V16I3.11, 26867284. 26867284

[38] Menajovsky M. F. , Espunyes J. , Ulloa G. , Calderon M. , Diestra A. , Malaga E. , Muñoz C. , Montero S. , Lescano A. G. , Santolalla M. L. , Cabezón O. , and Mayor P. , *Toxoplasma Gondii* in a Remote Subsistence Hunting-Based Indigenous Community of the Peruvian Amazon, Tropical Medicine and Infectious Disease. (2024) 9, no. 5, 10.3390/tropicalmed9050098, 38787031. PMC11125861 38787031

[39] Achaw B. , Tesfa H. , Zeleke A. J. , Worku L. , Addisu A. , Yigzaw N. , and Tegegne Y. , Sero-Prevalence of *Toxoplasma Gondii* and Associated Risk Factors Among Psychiatric Outpatients Attending University of Gondar Hospital, Northwest Ethiopia, BMC Infectious Diseases. (2019) 19, no. 1, 10.1186/s12879-019-4234-6, 2-s2.0-85068542058, 31272401. PMC6610991 31272401

[40] Cook A. J. C. , Gilbert R. E. , Buffolano W. , Zufferey J. , Petersen E. , Jenum P. A. , Foulon W. , Semprini A. E. , and Dunn D. T. , Sources of Toxoplasma Infection in Pregnant Women: European Multicentre Case-Control Study Commentary: Congenital toxoplasmosis---further thought for food, British Medical Journal. (2000) 321, no. 7254, 142–147, 10.1136/bmj.321.7254.142, 10894691. 10894691 PMC27431

[41] Jones J. L. , Kruszon-Moran D. , Wilson M. , McQuillan G. , Navin T. , and McAuley J. B. , *Toxoplasma Gondii* Infection in the United States: Seroprevalence and Risk Factors, American Journal of Epidemiology. (2001) 154, no. 4, 357–365, 10.1093/aje/154.4.357, 2-s2.0-0035881198. 11495859

[42] Stagno S. , Dykes A. C. , Amos C. S. , Head R. A. , Juranek D. D. , and Walls K. , An Outbreak of Toxoplasmosis Linked to Cats, Pediatrics. (1980) 65, no. 4, 706–712, 10.1542/peds.65.4.706. 7189277

