# Supplementary material for: Factors Associated With Toxoplasma gondii Infection Among Pregnant Women Admitted for Delivery in Selected Hospitals in Dar es Salaam, Tanzania
Source: Biomed Res Int. 2026 Jan 5;2026:8584162. doi: 10.1155/bmri/8584162 (PMC12766278; doi:10.1155/bmri/8584162)
Supplement: Supplementary file 1 — Supporting Information 1 Additional supporting information can be found online in the Supporting Information section. File S1: Questionnaire for toxoplasmosis—English version. [file BMRI-2026-8584162-s001.docx]

**Questionnaire for Toxoplasmosis - English Version**

Date ___/___/___

Hospital Reg Number: ________

Process number: _________________

Hospital Name:___________________

**SOCIAL DEMOGRAPHIC DATA**

**(Cycle the correct answer)**

1. What is your age in years? **________**
2. What is your highest level of education
3. No education
4. Primary level
5. Secondary level
6. College/University level
7. In which category is your residence?
   1. Rural
   2. Urban
8. What is your marital status?
9. Married
10. Never Married
11. Divorced/Separated
12. Widowed
13. What is your occupation?
    1. Formerly employed
    2. Housewife/Student
    3. Self-employed/farmer
14. Socioeconomic status
    1. Low
    2. Middle

**OBSTETRIC CHARACTERISTICS**

**(Cycle the correct answer)**

1. Gravidity
   1. Primigravidae
   2. Multipara
2. How many times have you visited antenatal care during this pregnancy?……….
3. Have you ever had an abortion?
   1. Yes
   2. No

**RISK PROFILE**

**(Cycle the correct answer)**

1. Do you have contact with cats other than yours?
2. No
3. Yes
4. Do you own a cat?
   1. No
   2. Yes
5. Do you consume rodents for food?
6. No
7. Yes
8. Do you eat salad?
   1. No
   2. Yes
9. Do you eat raw or undercooked food?
   1. No
   2. Yes
10. Do you consume unpasteurized milk or dairy products?
    1. No
    2. Yes
11. Do you eat soil?
    1. No
    2. Yes
12. Do you have a habit of consuming game meat such as birds, rabbits, wild boars etc?
    1. No
    2. Yes
13. What type of water do you drink at home?
    1. Treated/Bottled water
    2. Untreated water
